# Identification of damage development in the core of steel cord belts with the diagnostic system

**DOI:** 10.1038/s41598-021-91538-z

**Published:** 2021-06-11

**Authors:** Ryszard Blazej, Leszek Jurdziak, Agata Kirjanow-Blazej, Tomasz Kozlowski

**Affiliations:** 1 Faculty of Geoengineering, Mining and Geology, Wroclaw University of Science and Technology, 27, Wybrzeże Wyspiańskiego st., 50-370 Wrocław, Poland; 2 Faculty of Electronics, Wroclaw University of Science and Technology, 27 Wybrzeże Wyspiańskiego st., 50-370 Wrocław, Poland

**Keywords:** Engineering, Coal

## Abstract

Belt conveyors are used for transporting bulk materials over distances. The core of the belt, by transferring the longitudinal stresses and ensuring proper frictional coupling of the belt, enables belt movement and transportation of materials on its surface. As the belt cover and edges are used, the belt becomes abraded, and the core is subject to fatigue. The result is the development of cracks in rubber covers across the belt, which leads to the development of damage not only along the cables (the natural direction of water migration and corrosion) but also in the direction transverse to the belt axis. Conducting a series of scans of the St-type belt operating in one of the underground copper ore mines in Poland allowed identifying the number of failures as well as their size and changes over time. These data were in turn used to determine the measures defining the condition of the belt such as the density of defects (the number of defects per 1 m of the belt), the density of the area of damage (the area of damage per 1 m of the belt) and the change in the average area of a single defect over time. By determining the regression of these measures in time and the rate of damage development in both directions (along the axis of the belt and across the belt), it was possible to forecast future states of the belt, as well as to evaluate the costs of different belt replacement strategies and the economic rationalization of the decision to replace them. This research has become possible owing to the development of the DiagBelt system for two-dimensional imaging of the damage to the core of steel-cord belts with resolution sufficiently high to allow tracking the development of single core defects.

## Introduction

Belt conveyors are used for economical transportation of bulk material along distances of up to 100 km. Individual conveyors can reach lengths of up to 20 km. Bulk material rests on the surface of a troughed belt forming a closed loop. The conveyor belt is an elastic rubber tape with a reinforced core transferring longitudinal stresses and ensuring a frictional coupling necessary to ensure the movement of the belt and to overcome the resistance to movement. In long conveyors, the core must be sufficiently strong, so it contains parallel steel cables vulcanized in the core rubber with high adhesion to the cords. The core is protected against damage by rubber covers resistant to abrasion and punctures. Sometimes the core is additionally protected against punctures with a breaker, i.e. a protective layer (textile or cross-linked cables). Over years of its operation, a conveyor belt is subject to various degradation processes. Some of them are the consequence of its friction with the structural elements of the conveyor and the transported spoil. Others are fatigue-related due to the process of its bending on drums and rollers, which leads to the weakening of belt joint sections in the loop and thus to the weakening of the core structure. Some damage to the covers and the belt core remains hidden and is related to punctures from sharp-edged lumps at the loading points of the spoil onto the belt. The structure of the covers and the core is subject to destruction in a degree dependent on the energy of falling lumps, as well as on the safeguards applied in the construction of hoppers, on other devices used to absorb the energy of the falling material or even on changes in the construction of the belt (the use of protective layers—breakers). Cord damage identified with the DiagBelt magnetic system is most often associated with the puncture of the cover penetrating to the cable. Through these discontinuities, aggressive mine water leeks into the core, causing the corrosion of cables, which spreads along the cords (in the direction of the belt axis). Long-term research on changes in the size of single cord defects in one of the underground ore mines showed that damage also increases across the belt. Most likely caused by discontinuities in the covers, the original defects initiate the propagation of cracks across the belt due to its bending on the drums and rollers. The article analyzes the rate of these changes in time both in the direction of the belt axis and in the direction transverse to it. The results indicate that the size of a single defect is greater in the transverse direction than along the belt axis. This paper attempts to quantify these changes.

Magnetic diagnostic systems have been used to evaluate the technical condition of conveyor belts since early 1970s^1–4^. Originally, they generated an analog signal, which was aggregated for measuring circuits (belt strips) several tens of centimetres in width, scanned sequentially with one measuring head during several cycles, or measured across the entire belt width at once owing to several measuring heads installed across the belt. This technique did not allow the identification of individual defects several centimetres in size.

Modern, miniaturized diagnostic tools allow up to 200 coils to be installed in the measuring head (bar) which thus covers the entire width of the belt with the goal to identify defects to single cords. Instead of analog signals, they generate a digital signal consisting of discrete zeros and ones which correspond to the changes of the magnetic field, enabling 2D imaging of defects. Such devices allow easier identification of the location and size of the damage in the belt core. The results of belt loop scans performed on several occasions in one of Polish underground mines served to develop a model of damage development in the core of steel cord conveyor belts^5^.

## Method for steel cord defect detection in conveyor belts by the observation of changes in the magnetic field of magnetized cords

Defects in the core of steel cord belts are identified by registering and analysing changes in the magnetic field of magnetized cables around their defects, e.g. cutting cables or their cuts, their crushing and corrosion. In the analysis of damage to hoisting ropes, special wire breaks can be identified. In the case of conveyor belts, cuts of several cables and their corrosion in the same cross-section of the belt are more critical. This type of damage weakens the strength of the belt, which may cause the belt loop to break. Corrosion of lines on a large surface can lead to holes in the belt, spillage of spoil or an emergency stop of the conveyor and as a result entail high costs of damage removal and significant production losses. The use of a magnetic bar which has a large number of sensors and offers a dense, two-dimensional image of core damage (as in the case of DiagBelt) allows tracking both the changes of defects in individual cables and damage development over time. Every significant defect in the core increases over time. Repetitions of the belt scanning process allow detecting these changes and building a model of the rate of damage development in both directions: along the axis of the belt (along the cables) and in the transverse direction. Damage development in this direction is particularly exciting, because it causes the development of cracks in the rubber, initiated by punching the covers into the core or out of it. During the bending of the belt on the drums and on the idler sets, the cracks increase, and the water begins to reach adjacent cables, causing corrosion. That is why it is so important to repair major punctures in order to delay the cracking process.

Development of cracks in rubber and adjacent steel cables corrosion requires further modelling similar to FEM analysis of the effect of radial expansion and crack width determination of corroded reinforcement of concrete^6,7^ and damage modelling for elastic and viscoelastic materials at large strain^8^. Application of DiagBelt for the first time allowed detection and measuring of similar phenomenon taking place in rubber belts.

**Figure 1:**
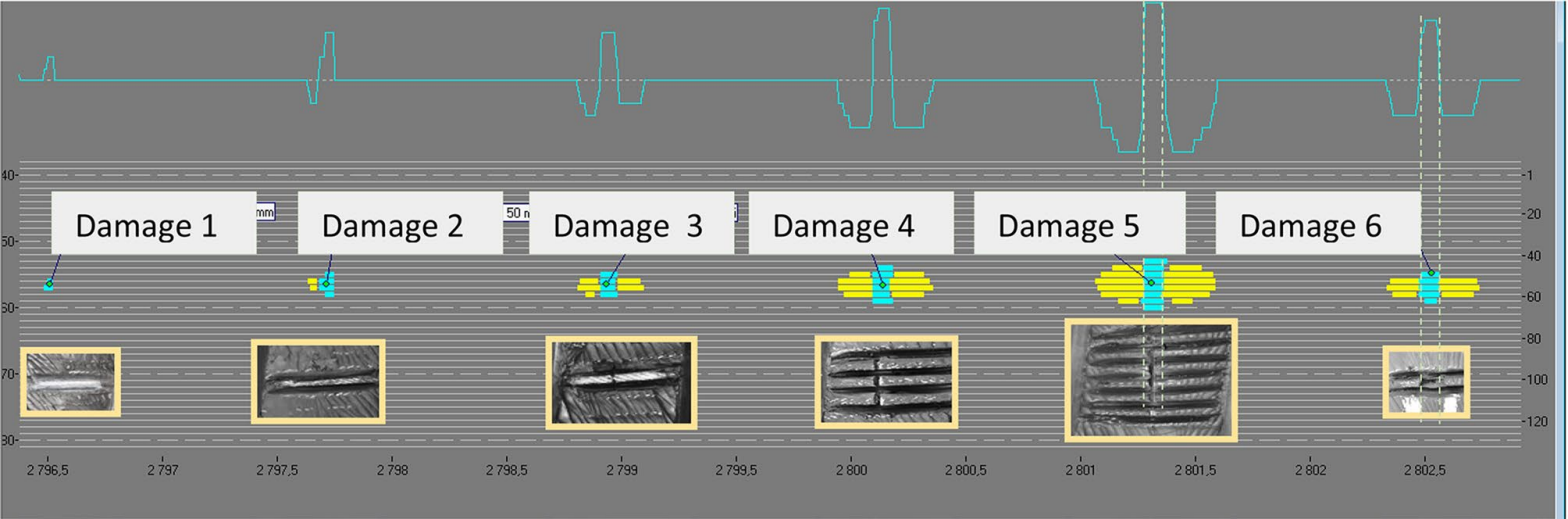
Condition of a belt section between joints with actual defects and their locations indicated (internal documentation)^12^ (screenshot of Belt Analysis software version 0.6, http://www.beltscan.com/).

The DiagBelt system has been described in a number of publications (e.g.^9–11^). Figure 1 shows the method of damage imaging with the use of the BeltGuard probe confronted with an image of a significant belt defect^12^.

**Figure 2:**
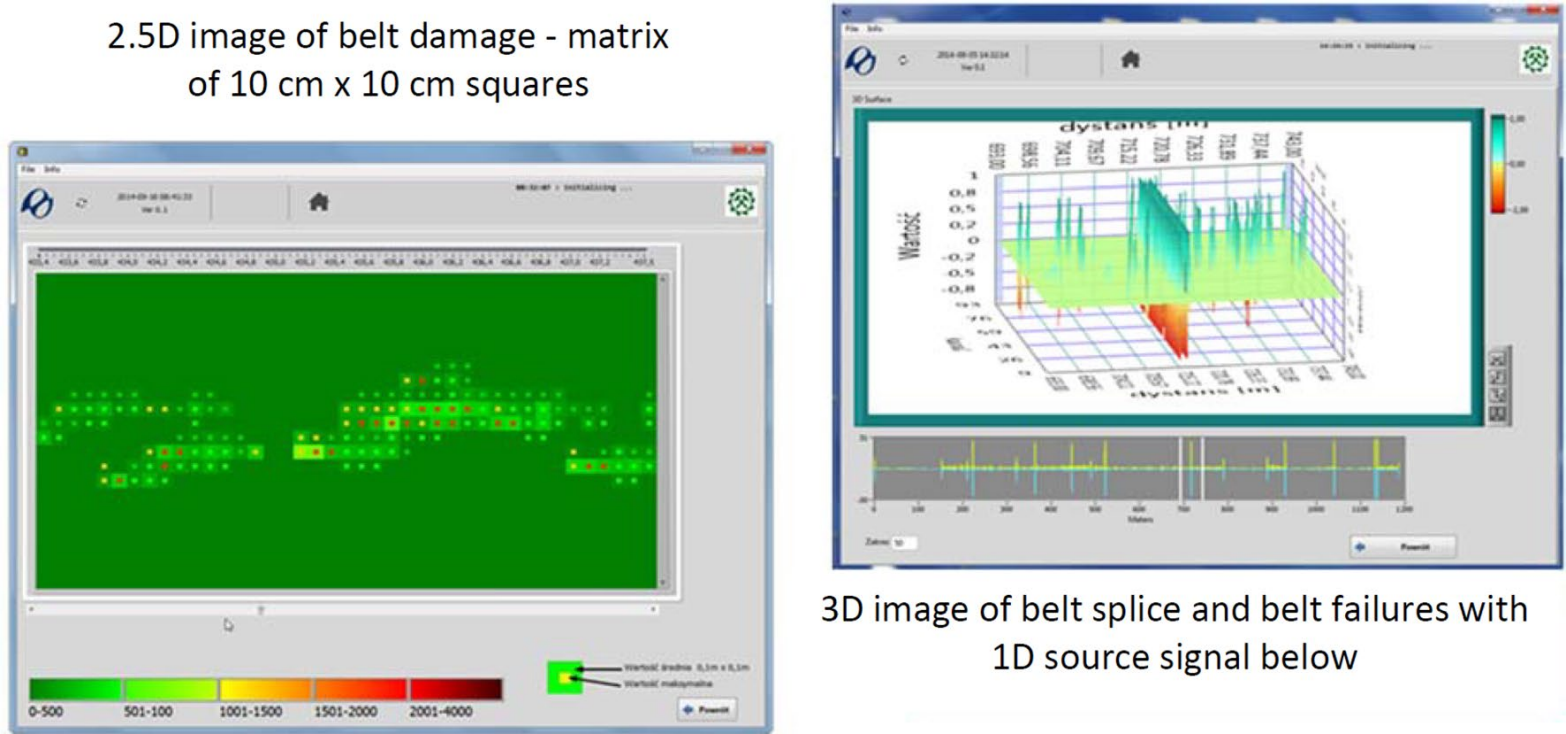
Various methods for imaging defects in the core and in the splice: 2.5 D matrix 10 × 10 cm (left) and 3D and 1 D signal (top right and bottom right)^12^ (screenshot of DiagBelt proprietary software, version 1.0, http://diagbelt.pwr.edu.pl/).

As the system is of high resolution and offers the possibility to analyze field changes on various sensitivity levels, it allows 3D damage visualizations (Fig. 2, on the right), and their aggregation to squares 10 cm × 10 cm provides a 2.5 D image, in which the background color represents mean signal intensity level and the central point represents the maximum for the area (Fig. 2, on the left).

**Figure 3:**
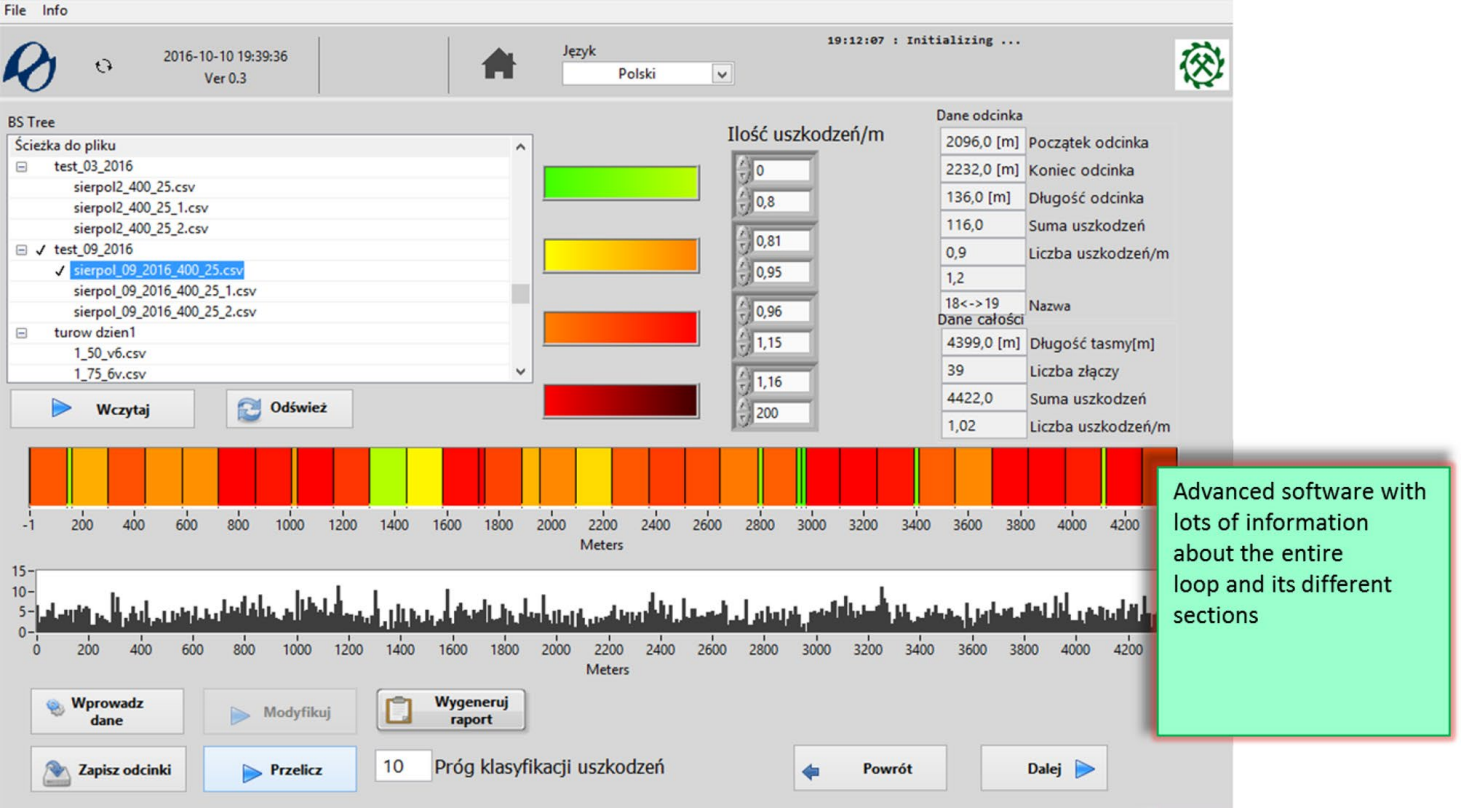
Belt loop on a conveyor viewed in DiagBelt with averaged and color-coded identification of the condition of successive belt sections and with a histogram of damage density along the belt axis (screenshot of DiagBelt proprietary software, version 1.0, http://diagbelt.pwr.edu.pl/).

The system has sufficient resolution to allow a precise inventory of defects and the representation of aggregated damage level measures, such as for example damage density and area per running meter and the damage histogram on the belt section for a particular belt fragment or for the complete belt section from splice to splice^11^. Aggregating numerous defects into a synthetic measure is necessary to evaluate the general condition of a belt section, which is coded with colors (Fig. 3) enabling the user to instantly evaluate the degree of wear and tear in each of the segments in the loop. Green color corresponds to no damage and red color indicates a significant number of defects which may pose a threat to the functioning of a transportation system (Fig. 3).

**Figure 4:**
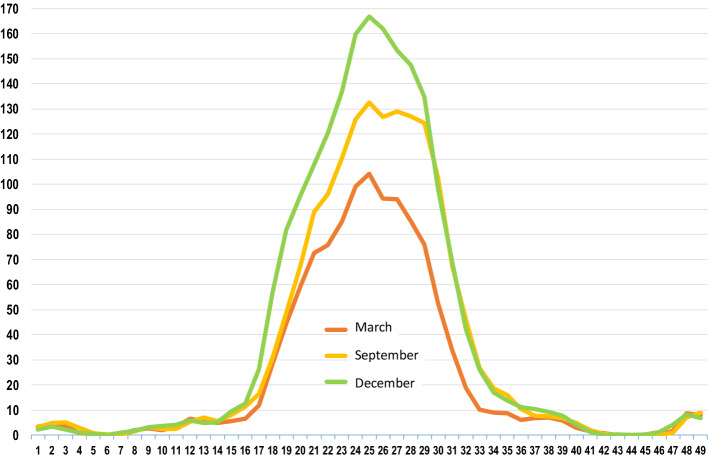
Histogram of average damage density in the cross-section of the belt, for 4 sections analyzed during 3 measurements in 2016^20^, (Microsoft Excel, version 2010, own data).

Visual representation of belt sections and their fragments allows both the scope of each defect and the damage density to be identified along the belt axis and in the cross-section (Fig. 4), and also provides information on the locations in need of repair (Figs. 1, 2, 3, and 4). Owing to the system’s resolution, the size of each defect can be estimated and tracked over time^13^.

The possibility to analyze the distribution of belt core defects in the cross-section of the belt represents a significant improvement. Several research centers (e.g. Wroclaw University of Science and Technology^14,15^, and in Košice^16,17^) conduct intensive studies into belt puncture resistance. The investigations include the influence of the energy and shape of material lumps on the type of damage to the belt and its core^18^. These are also classified^19–21^ with the purpose to increase belt puncture resistance by modifying the belt support^22,23^, the belt design^14,24,25^, the composition of the rubber mixture^18^ or the introduction of breakers. The magnetic system^26^ or other tools for evaluating the effects of punctures^27^ allowed a non-invasive inspection of cores and belts subjected to punctures. Typically, puncture resistance tests are performed in laboratory conditions. Until present, no possibility existed to identify damage distribution in the cross-section of the belt. The DEM software^28–32^ enables evaluations of how the shape of the hopper or the transfer chute influences the energy of the lump and the drop locations^33–35^, as well as wear processes in both the belt covers and the transfer devices^36,37^. However, it was impossible to verify this data in practice—a procedure of great significance in the validation of simulation models. From the user's perspective, damage distribution should be importantly even and not concentrated in the central part (Fig. 4), as in such case the belt wears faster in the central part while its edges or side parts practically remain in good condition. A belt which is evenly worn in its central part cannot be refurbished and therefore its core cannot be reused (two or even three times). Belt refurbishment consists in milling its worn external covers and in re-vulcanizing new covers with the old core. As such, the procedure meets the conditions of circular economy^38–40^. Disposal of the belt due to its overly worn central part is considered wasteful, as a large part of the belt is still in operating condition. As can be seen in Fig. 4, more than 50% of the belt cross-section remains in good condition.

## Results of measurements with the Diagbelt diagnostic system in an underground mine

The data on belt condition collected during a series of five consecutive belt scans (Table 1) and the identification of the number and size of cord defects allowed a statistical analysis of the rates of damage growth over time in both directions. The rate of new damage formation was also determined. Submission of these two processes allows forecasting future belt condition. For this purpose, specific measures of belt wear rate were introduced, such as the belt damage density (the number of defects per 1 m of belt), the density of belt defect area (the area of defects per 1 m of the belt) and the change in the average area of one defect over time. By determining the regression of these measures in time and the rate of damage development in both directions (along the axis of the belt and across the belt), it was possible to forecast future states of the belt, as well as to evaluate the costs of different belt replacement strategies and the economic rationalization of the decision to replace them.

**Table 1:** Dates of the measurements.

Scan number	Scan date	Belt age in months	Number of sections
Date of installation	Feb. 2011	0	32
1	Mar. 2016	60	39
2	Sept. 2016	66	39
3	Dec. 2016	69	40
4	Mar. 2018	84	44

**Figure 5:**
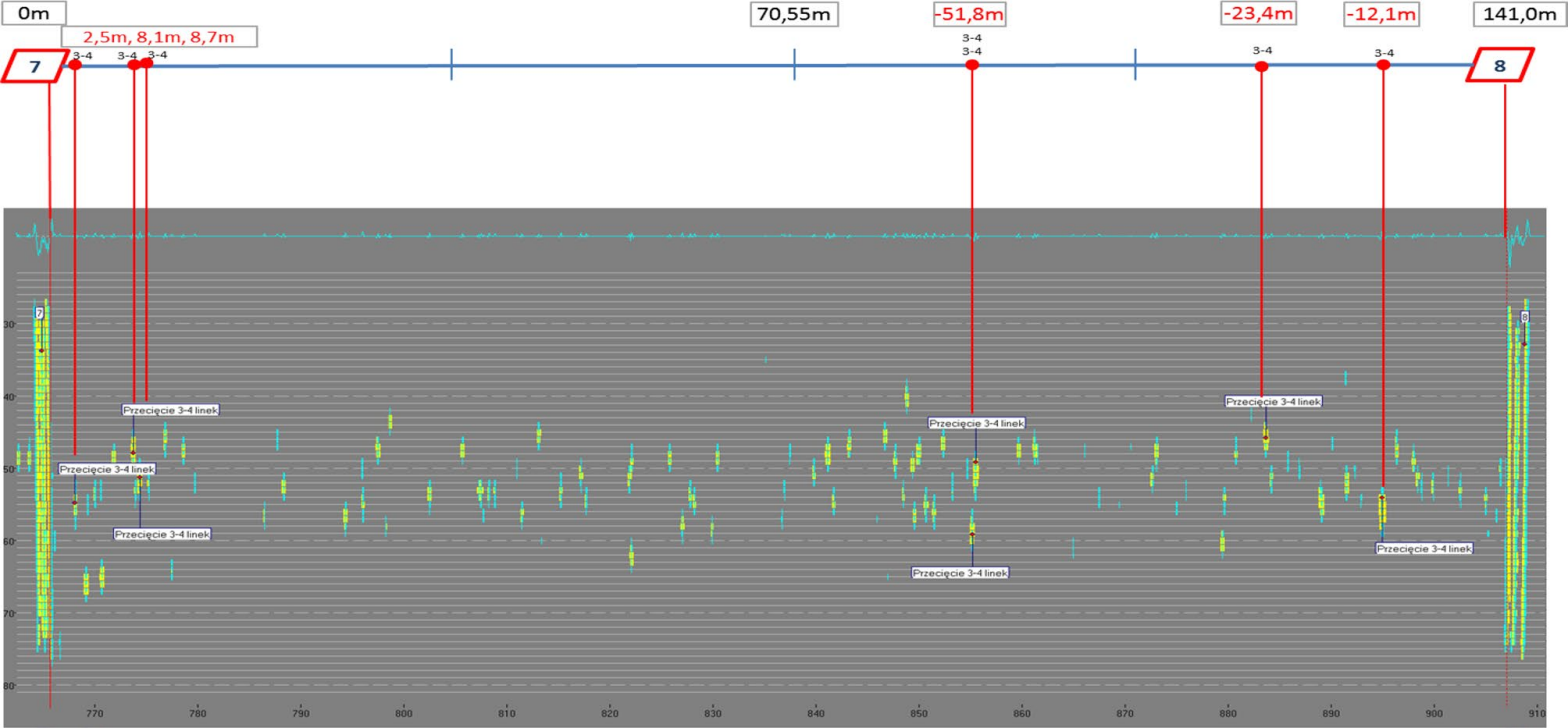
2D representation of a belt section after 86 months in operation^5,42^ (screenshot of DiagBelt proprietary software) (screenshot of Belt Analysis software version 0.6, http://www.beltscan.com/).

The diagnostics of the loop of steel cord belt operated in a Polish underground mine on a conveyor 2200 m in length performed in order to evaluate the condition of its core provided results which served to demonstrate random belt degradation process and its change over time. The first inspection of belt St 3150, 1200 mm in width, was performed in March 2016, when the belt had been operated in the mine for 60 months (5 years). The tests were repeated after 6, 9 and 24 months. This allowed observations of changes in belt damage condition. The evaluation covered the technical condition of the core of steel-cord belt used in the transportation of copper ore. Figure 5 shows the condition of a section of the inspected loop and its indicated damage. The diagnostic system was located in the vicinity of the conveyor’s head station, on the belt’s flat section. Two magnet heads and a measurement head were installed at a distance of 40 mm above belt cover. During the entire test period, the belt speed, as measured with a tachometer, was approx. 2.5 m/s. The measurement data were exported to separate *.CSV (Comma-Separated Values) files to facilitate their further processing and the statistical analysis of the defects.

The identified defects are indicated by a change of colour from yellow to blue (change of the magnetic field from negative to positive) (Fig. 5). The total of the aggregated source signals from all sensors is shown above as a blue signal line in the grey background. Splices (7 and 8) are visible on both ends of the belt section. The required repairs are indicated above, along with their precise locations measured from the left splice in meters and with information on the number of defective cables.

The exported digital data regarding the condition of the belt section and the defects were aggregated by calculating the total number of defects per successive running meters of the inspected belt section between splices 7 and 8. The length of the belt section was 138 m. Thus, aggregated belt condition measure was obtained—damage density (GUi). Damage density can be used as a basis to evaluate the technical condition of a complete belt section or of its individual parts. In this case the analysis covered successive 1-m fragments (segments) of the inspected section. In the case of segments one meter in length, the density corresponds to the number of defects identified in the areas of successive segments (GUsi = Nsi). In this part, the analysis focused on changes in damage density distribution along belt axis during consecutive scans.

**Figure 6:**
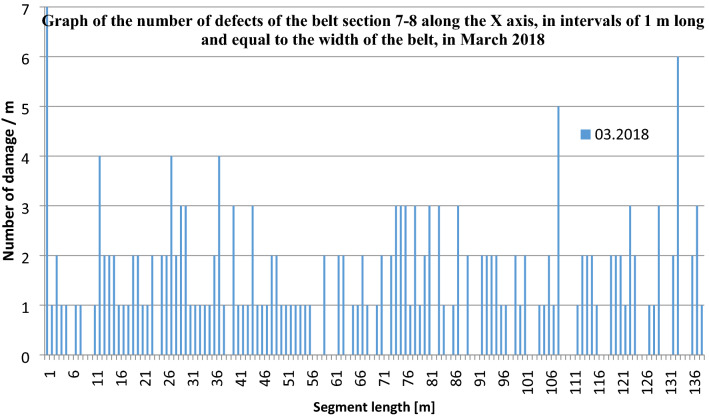
Damage density in 1-m-long belt segments along belt axis, for the analysed belt Sect. 7–8—state of March 2018 (4th measurement)^5,42^ (Microsoft Excel, version 2010, own data).

**Figure 7:**
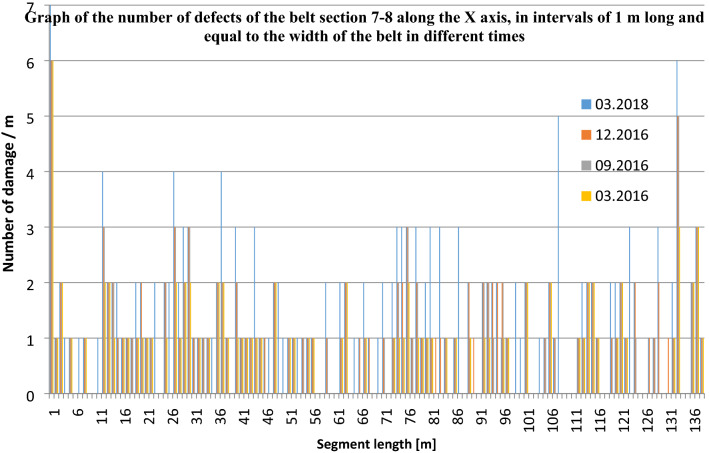
Number of defects in 1-m segments of the belt section along its axis (X direction) in consecutive scans from March, September and December 2016, as well as from March 2018^5,42^ (Microsoft Excel, version 2010, own data).

The numbers of defects (their densities) are natural numbers. Zero represents no defects and n represents n defects identified in a particular 1-m segment. This is shown in the graph of Fig. 6. The graph in Fig. 7 shows the change of damage density over time in consecutive scans of the 7–8 section from March, September and December 2016, as well as from March 2018. As can be seen, even after many years in operation, some 1-m belt sections still show no trace of damage. Some other fragments, on the other hand, reach 7 defects per 1 m.

In order to investigate whether defects along the axis of the belt section are randomly distributed, changes of the number (density) of defects in consecutive 1-m segments were analyzed for randomness in a series of tests.

**Figure 8:**
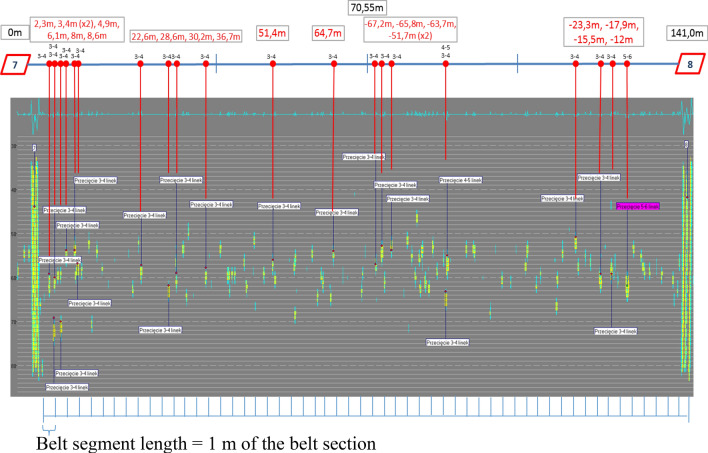
Two-dimensional image of core damage in the belt section with 1-m segments, which served to calculate damage—state of March 2018^5,42^ (screenshot of Belt Analysis software version 0.6, http://www.beltscan.com/).

The calculations of the number of defects in consecutive segments were performed by analyzing the defects identified in the digital image of magnetic field changes in the area of the 7–8 belt section, in its consecutive 1-m segments (Fig. 8).

## Randomness tests of changes in the number of defects in 1-m segments of the belt section

Intuition suggests that belt core defects are distributed randomly in the direction of belt axis. Regardless of the intensity of the material stream fed to the conveyor belt, the probability of damage to each 1-m segment in the section and in the entire belt loop is identical. It is hardly possible that the relatively short belt loop travel cycles around the conveyor (one cycle lasts several tens of minutes) could be correlated with the changing intensity of the bulk material stream fed to the conveyor or with the frequency of large lumps of material being dropped on the belt (with energies exceeding critical values) over a belt operation period counted in years. Each 1-m segment of the belt passes the feed points as many times as the entire belt loop with the accuracy limited only by single rotation cycles. These insignificant differences counted in single cycles (and resulting from the changes in the starting and stopping of the conveyor, as well as from cyclical feeding of material on the conveyor in copper ore mines) should be eliminated over many years of belt operation. The belt loop needs approximately 30 min to perform a full cycle around the conveyor, and during one working shift conveyors are operated for several hours and perform more than 10 cycles. During a week, the number of cycles reaches approx. 250, and during a month—more than a thousand. After a year, the belt loop performs approx. 12 000 cycles, and after 5 years—60 000 cycles. Slight differences which occur during the starting and stopping of the conveyor, or when the material starts and stops to be fed should be completely eliminated over a period of many years. Any correlation between the segment location in the loop and moments when the material is fed to it (cyclical loading) is improbable.

It is indeed possible in one cycle, and cyclical changes of the fed material stream could significantly influence the number of lumps falling on a particular segment in the section and in the loop. However, momentary changes of material stream intensity^41^ are importantly not equivalent to the resulting cord damage, and the belt loop performs thousands of such cycles during its operation. Therefore, the recorded defects are the result of multiple interactions between the material lumps and the belt and of its movement with the transported material over a great number of kilometers along the belt route. In a year, the belt on the investigated conveyor transports mined material over a distance of 25 000 km, so slight differences which occur during one cycle (in which the material is transported along a distance of 2.2 km) should be eliminated. Obviously, intuition requires formal verification and therefore the defects in the consecutive segments were subjected to appropriate randomness tests.

**Figure 9:**
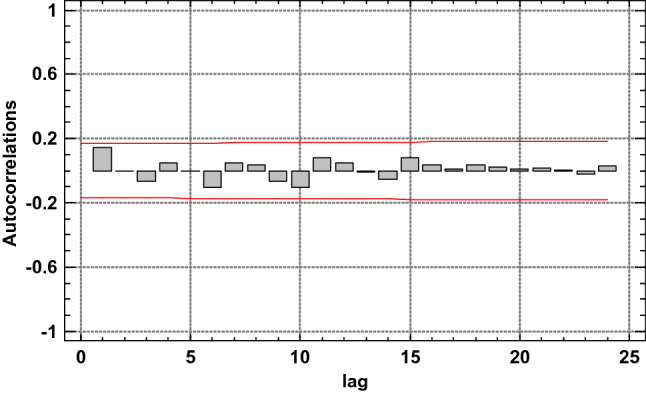
Estimated autocorrelations for various lags for the number of defects (density) in 1-m segments of the 7–8 belt section, for the 1st measurement^5,42^.

The graph in Fig. 9 shows estimated autocorrelations of the numbers of defects in segments (their density) for 24 lags. In this case delay means a geometric lag by one meter along the belt section axis. The autocorrelation coefficient for lag k is the measure of correlation between the number of defects in a segment in position t and in a position shifted to t-k. The figure also shows the 95% confidence interval around 0. If the autocorrelation for a given lag exceeds this limit, then a statistically significant correlation exists for this lag with 95% confidence level. No statistically important autocorrelations were identified for the first measurement of the condition of the 7–8 belt section, which suggests that the number of defects in 1-m segments is completely random and constitutes the so-called white noise.

**Figure 10:**
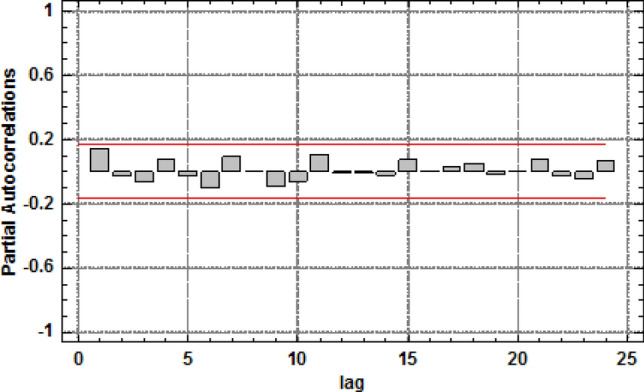
Estimated partial autocorrelations for various lags for the number of defects (density) in 1-m segments of the 7–8 belt section, for the 1st measurement^5,42^ [Statgraphics Centurion Version 18.1.06 64-bit version (academic license of Wrocław University of Science and Technology), https://www.statgraphics.com/].

The graph in Fig. 10 shows estimated partial autocorrelations between the number of defects in 1-m segments of the belt section for various lags. The partial lag autocorrelation coefficient k is a measure of the correlation between the number of defects in the t and t + k location with allowance for the correlation for all shorter lags. It may serve to evaluate the sequence of the autoregression model required to fit the data. The figure also shows the limits of 95.0% confidence intervals around 0. The fact that the estimated coefficients remained within the limits for all of the 24 lags indicates that no statistically important partial correlations exist at the 95% confidence level.

**Figure 11:**
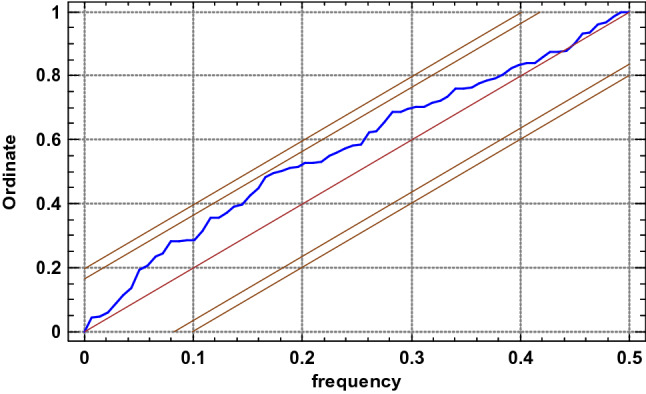
Integrated periodogram for the number of defects (density) in 1-m segments of the 7–8 belt section along its axis for 1st scan^5,42^ [Statgraphics Centurion Version 18.1.06 64-bit version (academic license of Wrocław University of Science and Technology), https://www.statgraphics.com/].

**Figure 12:**
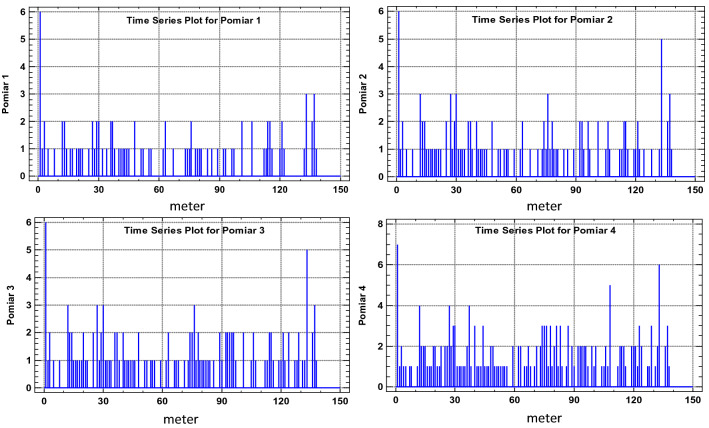
Number of defects (damage density) for consecutive 1-m segments of the 7–8 belt Sect. 138 m in length, in consecutive scans^5,42^ [Statgraphics Centurion Version 18.1.06 64-bit version (academic license of Wrocław University of Science and Technology), https://www.statgraphics.com/].

**Figure 13:**
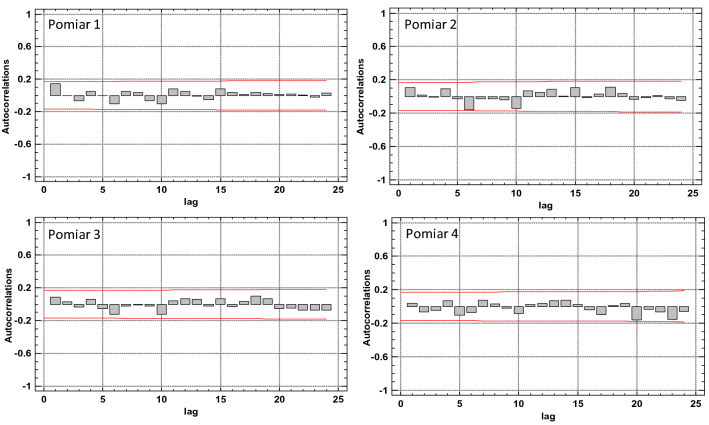
Estimated autocorrelations for various lags for the number of defects (density) in 1-m segments of the 7–8 belt section, for the consecutive scans^5,42^ [Statgraphics Centurion Version 18.1.06 64-bit version (academic license of Wrocław University of Science and Technology), https://www.statgraphics.com/].

**Figure 14:**
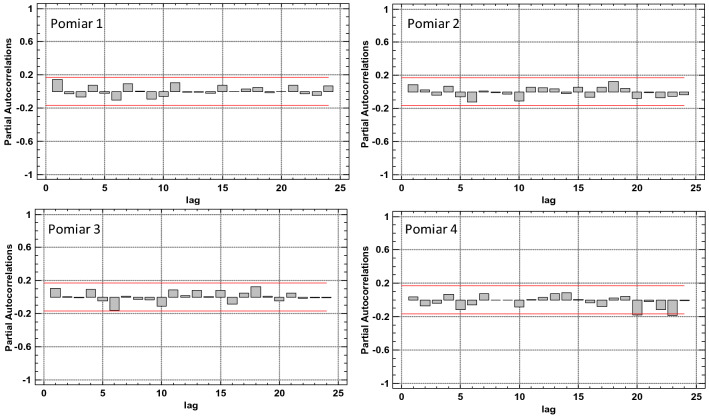
Estimated partial autocorrelations for various lags for the number of defects (density) in 1-m segments of the 7–8 belt section, for the consecutive scans^5,42^ [Statgraphics Centurion Version 18.1.06 64-bit version (academic license of Wrocław University of Science and Technology), https://www.statgraphics.com/].

**Figure 15:**
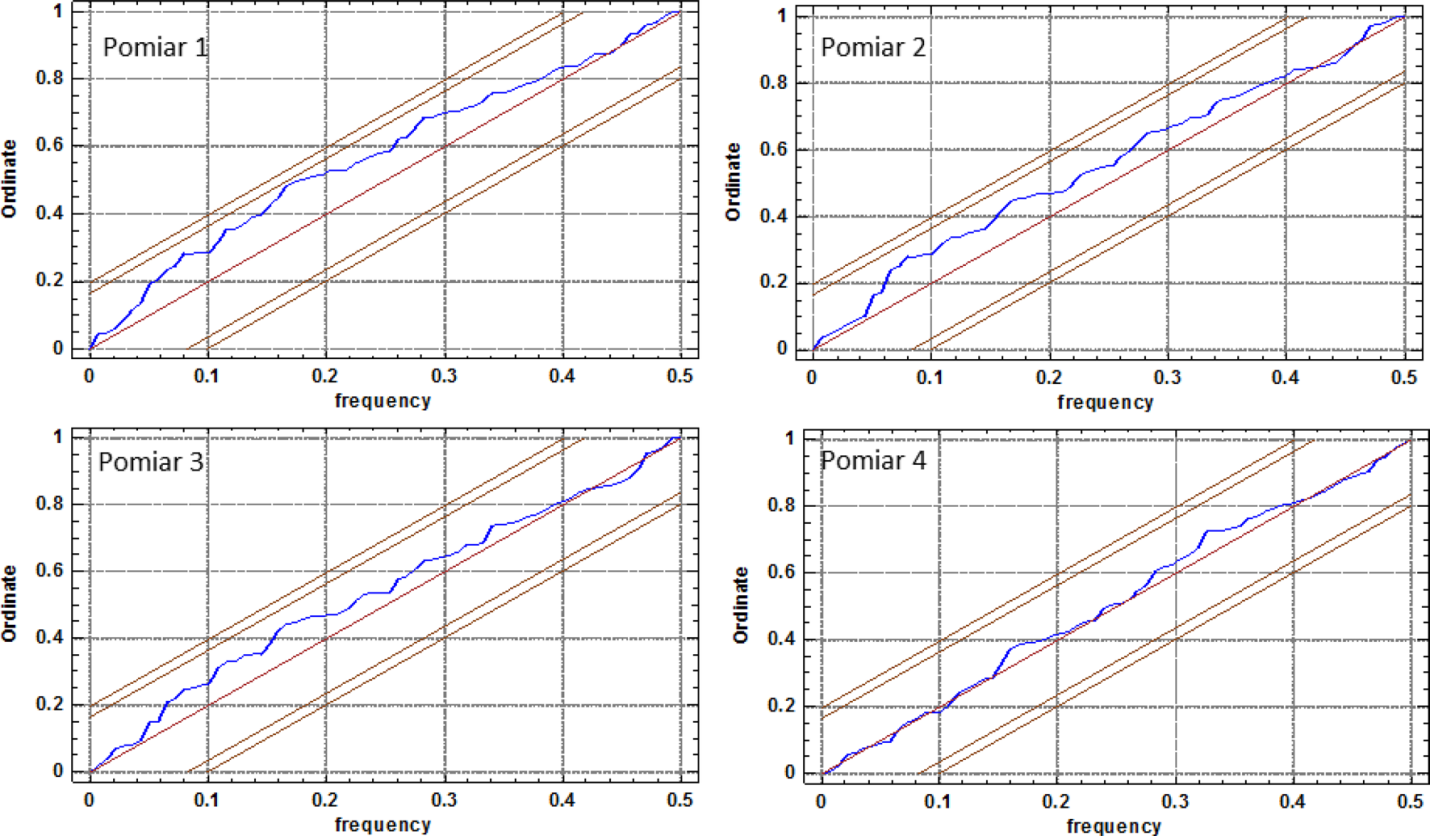
Integrated periodogram for the number of defects (density) in 1-m segments of the 7–8 belt section along its axis for consecutive scans^5,42^ [Statgraphics Centurion Version 18.1.06 64-bit version (academic license of Wrocław University of Science and Technology), https://www.statgraphics.com/].

The integrated periodogram (Fig. 11) represents an accumulated total of the periodogram ordinates. It also shows the 95% and 99% Kolmogorov–Smirnov limits. If damage densities of the sections in the 1^st^ measurement are only a random series of values, then the integrated periodogram should remain within the strips around the diagonal line. The fact that the plotted line remains within the 95% limits makes it impossible to reject the hypothesis that the series of damage densities in consecutive segments is random at a 95% confidence level. Similar results were obtained for the number of defects in segments, their autocorrelations and partial autocorrelations, as well as periodograms for consecutive scans, as illustrated in Figs. 12, 13, 14, and 15.

The analysis of the measurements along axis X as a time series (analysis of the autocorrelation and partial autocorrelations, as well as plotting the periodograms) demonstrated that no atypical fragments exist along the axis of the section and each consecutive meter of the belt is subjected to similar, random damage. The process of damage formation along the belt axis is thus random and represents white noise. The cyclical load changes on belt conveyors operated in copper ore mines^3^ have not translated into the cyclical character of belt damage along its axis. The defects are therefore caused by accumulated interaction between the transported material and the belt during many operating cycles. The cyclical changes of load feeds onto the belt is eliminated as the belt passes in tens of thousands cycles under the two feed points. Therefore, the identifiable time-related cyclical character of changing load exerted on the belt does not translate into the cyclical character of damage distribution along the belt axis (the distribution of the number and locations of defects along the axis of the belt loop).

## Conclusions

The development of measures to assess the degree of belt wear and the rate of development of belt defects over time allows more rational belt management. By including the limit states of the level of these measures into the belt-replacement policies, it is possible to forecast corrective actions, and indicate fragments of belts in need of replacement, as well as determine the right moments for replacing the entire belt sections. Owing to this, the stock of spare belts can be reduced, as the stream of belt demand over time can be forecast. Taking into account costs allows the user to determine and forecast future costs—the budget necessary to ensure continuous operation of the belt conveying system. The use of the identified functions in the simulation allows determining the costs of various belt replacement strategies and thus finding a strategy that will ensure minimized unit transport costs.

Changing the belt damage representation from a one-dimensional signal to a 2D signal (Figs. 1, 2 and 5) allows for quick data interpretation and belt condition assessment. Damage concentrations that pose a risk to the continuous operation of the belt loop on the conveyor can be clearly identified. Users can independently estimate the number, scale and location of defects. The analysis of one-dimensional signals (Fig. 1) from many measurements requires a trained specialist, and detailed interpretation is time-consuming. It is very difficult or practically impossible to quantify the size of a single defect and observe its changes over time.

The digital recording (− 1, 0, + 1) of changes in the magnetic field corresponding to the defects enables the compression of the resulting description of the belt condition (by removing nonsensical zeros from the record) and the storage of data from subsequent scans of the same belt loop for individual conveyors. By registering these changes at different sensitivity levels, it is possible to assess not only the location and size of damage, but also its intensity, and then visualize it on 2D and 3D charts (Fig. 3).

Records in digital form allow uncomplicated quantification of belt condition by introducing such measures as damage density per 1 running meter and further statistical analysis of the acquired data related to the belt’s condition.

The analysis of the aggregated changes in the level of damage in the belt section inspected at four points in time showed that the number of defects increases with time. The rate of damage development increased—it is an observation confirmed by the fact that in the first period, the monthly increment was at an average of 0.35 defect per 1 running meter of belt, and in the second period the monthly increment was at an average of 0.8. As can be seen, the development was twice greater.

The autocorrelation of damage density along the belt axis did not show any significant spatial relations. The uniform distribution provides an adequate description of the damage density distribution along the belt axis. The variability is very large and the coefficient of variation increases with the running time of the belt.

The development of corrosion cracks in rubber and adjacent wire ropes requires further modeling. Application of the diagnostic system for the first time made it possible to detect and measure unexpected phenomena occurring in rubber strips. The results of further modeling, testing and simulation can now be verified in practice with subsequent DiagBelt scans.
